# Multiple Gene Polymorphisms Associated with Exfoliation Syndrome in the Uygur Population

**DOI:** 10.1155/2019/9687823

**Published:** 2019-05-02

**Authors:** Yi-Nu Ma, Ting-Yu Xie, Xue-Yi Chen

**Affiliations:** Department of Ophthalmology, The First Affiliated Hospital of Xinjiang Medical University, Urumchi 830011, Xinjiang, China

## Abstract

**Background:**

Our previous data suggested that three single-nucleotide polymorphisms (SNPs), rs1048661, rs3825942, and rs2165241, of the lysyl oxidase-like 1 gene (*LOXL1*) are significantly associated with exfoliation syndrome (XFS) and exfoliation glaucoma (XFG). The following study investigated other SNPs that potentially effect XFS/XFG.

**Methods:**

A total of 216 Uygur patients diagnosed with XFS/XFG, and 297 Uygur volunteers were admitted to the First Affiliated Hospital at Xinjiang Medical University between January 2015 and October 2017. Blood samples were collected by venipuncture. Alleles and genotypes of *LOXL1*, *TBC1D21*, *ATXN2*, *APOE*, *CLU*, *AFAP1*, *TXNRD2*, *CACNA1A*, *ABCA1*, *GAS7*, and *CNTNAP2* were analyzed by direct sequencing.

**Results:**

The allele G of rs41435250 of *LOXL1* was a risk allele for XFS/XFG (*P* < 0.001), whereas the allele G of rs893818 of *LOXL1* was a protective allele for XFS/XFG (*P* < 0.001). After adjusting all data for age and gender, the following results were obtained: the frequency of genotype CC for rs7137828 of *ATXN2* was significantly higher in XFS/XFG patients than in controls (*P* = 0.027), while no significance was found with reference to the frequency of genotype TT. The frequency of genotype GG for rs893818 of *LOXL1* (*P* < 0.001) and the frequency of genotype AA were both significantly higher in XFS/XFG groups compared to the control group (*P* < 0.001). In addition, the frequency of genotype TT for rs41435250 of *LOXL1* was higher in XFS/XFG patients than in controls (*P* = 0.003), while no significant difference was found with reference to the frequency of genotype GG after adjusting for age and gender. In addition, the haplotypes G-A/T-G/G-G for rs41435250 and rs893818 were significantly associated with XFS/G.

**Conclusions:**

With reference to *LOXL1*, the rs41435250 resulted as a risk factor and rs893818 as a protective factor for XFS/XFG in the Uygur populations. Meanwhile, the rs16958445 of *TBC1D21* and the rs7137828 of *ATXN2* have also shown to be associated with pathogenesis of XFS/XFG.

## 1. Introduction

Exfoliation syndrome (XFS) is an age-related, systemic, elastic microfibrillopathy characterized by deposition and progressive accumulation of a white, fibrillary, extracellular material affecting intraocular and extraocular tissues [1]. A recent study has suggested a high prevalence of XFS in the Uygur population [2, 3]. XFS is characterized by rapid progression, high resistance to medical therapy, and poor prognosis and may lead to exfoliation glaucoma (XFG), open-angle glaucoma, angle-closure glaucoma, and acceleration of cataract insensibly [4]. In China, especially in Xinjiang, many XFS/XFG patients lost their visual acuity due to the lack of medical treatment.

Genetic factors have an important role in XFS pathogenesis. Our previous data have suggested that three single-nucleotide polymorphisms (SNPs), i.e.,rs1048661, rs3825942, and rs2165241, of the lysyl oxidase-like 1 gene (*LOXL1*) were significantly associated with XFS and XFG [5]. Moreover, Yao et al. have discovered that rs4886467, rs4558370, rs4461027, rs4886761, and rs16958477 SNPs located in the *LOXL1* gene promoter region are risk factors for XFS [6]. In addition, many other SNPs, such as rs429358 and rs7412 located on apolipoprotein E (*APOE*) [7], rs2107856 and rs2141388 of contactin-associated protein-like 2 (*CNTNAP2*) [8], rs41435250 and rs893818 of *LOXL1* [9], rs16958445 of TBC1 domain family member 21 (*TBC1D21*) [10], rs7137828 of autosomal-dominant ataxin 2 (*ATXN2*), rs35934224 of thioredoxin reductase 2 (*TXNRD2*), rs11732100 of actin filament-associated protein 1 (*AFAP1*), rs2472493 of ATP-binding cassette subfamily A member 1 (*ABCA1*), rs9897123 of growth arrest-specific 7 (*GAS7*) [11], rs4926244 of calcium voltage-gated channel subunit alpha1 A (*CACNA1A*) [12], and rs2279590 of clusterin (*CLU*) [13], have been associated with XFS/XFG. Accordingly, the aim of this study is to investigate whether these SNPs also affect XFS/XFG.

## 2. Materials and Methods

### 2.1. Ethical Approval

The Ethical Committee of the First Affiliated Hospital of Xinjiang Medical University, China, approved this study. In addition, the informed consent was obtained from all participants after explaining the objective and nature of the study. The study was conducted in accordance with the Declaration of Helsinki.

### 2.2. Study Population

A total of 216 Uygur patients who were diagnosed as XFS/XFG and 297 normal Uygur volunteers who were admitted at the First Affiliated Hospital of Xinjiang Medical University, the First People's Hospital of Kashgar, and the Kuqa County Hospital between January 2015 and October 2017 were enrolled in this study. XFS was diagnosed based on the previously described approach [5]. In brief, XFS was diagnosed by exfoliation materials on the anterior lens capsule or on the pupil margin in either eye with dilation of the pupils. The inclusion criteria were the following: (1) IOP ≥22 mmHg in either eye; (2) glaucomatous changes on the optic disc, defined as a cup-to-disc ratio >0.7 in either eye or an asymmetric cup-to-disc ratio >0.2 or notching of the disc rim; and (3) characteristic glaucomatous visual field loss [14]. Patients with other causes of secondary glaucoma, such as uveitis, pigment dispersion syndrome, and iridocorneal endothelial syndrome, were excluded from the study. All study subjects were unrelated and received comprehensive ophthalmic examinations.

Peripheral blood samples (2-3 ml) were collected from each subject by venipuncture. Genomic deoxyribonucleic acid (DNA) was extracted from the whole blood using a Genomic DNA Extraction Kit (The Beijing Genomics Institute, Beijing, China). The SNPs (rs429358, rs7412, rs2107856, rs2141388, rs41435250, rs893818, rs16958445, rs7137828, rs35934224, rs11732100, rs2472493, rs9897123, rs4926244, and rs2279590) were amplified by photoconductive relay (PCR) and directly sequenced [7–13]. Two sets of primers were used for amplification by PCR.

Genotypes of these SNPs were determined by direct DNA sequencing, using a BigDye Terminator v3.1 Kit (Applied Biosystems, Foster City, CA) in a 3730XL capillary sequencer (Applied Biosystems). The sequences were analyzed by sequencing analysis software Chromas (Technelysium Pty Ltd., Queensland, Australia).

### 2.3. Statistical Analysis

Statistical analysis was performed using SPSS v17.0 software package (SPSS Inc., Chicago, IL). Hardy–Weinberg equilibrium (HWE) analysis was tested by using the *χ*^2^ test in SAS/Genetics v9.1 (SAS Institute Inc., Cary, NC, USA). The comparison of allelic and genotypic frequencies between the patient and control groups, as well as haplotype association analysis, was performed using a standard *χ*^2^ test. A *P* value <0.05 was considered statistically significant. Relative risk association was estimated by calculating odds ratios (OR) along with 95% confidence intervals (CI).

## 3. Results

A total of 216 Uygur XFS/XFG patients (case group) and 297 normal Uygur volunteers (control group) were included in the study. In the case group, there were 146 males and 70 females (average age: 68 years), while in the control group, there were 159 males and 138 females (average age: 62 year) (Table 1).

All SNPs underwent the Hardy–Weinberg equilibrium test before further data analysis. Besides rs7137828 that deviated from HWE (*P*=0.006) in the control group and rs35934224 that deviated from HWE (*P*=0.005) in the case group, other SNPs were all in line with the HWE Table 2.

The allele association analysis showed that the frequency of allele G of rs41435250 and rs893818 of *LOXL1* was significantly higher in XFS/XFG patients than in controls (rs41435250: *P* < 0.001, OR = 1.791, 95% CI: 1.334–2.405; rs893818: *P* < 0.001, OR = 0.423, 95% CI: 0.318–0.563), while no significant differences were found for other alleles (*P* > 0.05) (Table 3).

The genotype association analysis showed that the frequency of genotype AA for rs16958445 of *TBC1D21* was higher in XFS/XFG patients than in controls (*P*=0.033, OR = 5.481, 95% CI: 1.151–26.11), while the frequency of genotype GG was not significantly different between the two groups. After adjusting all data for age and gender, the following results were obtained: the frequency of genotype CC for rs7137828 of *ATXN2* was significantly higher in XFS/XFG patients than in controls (*P*=0.027, OR = 0.322, 95% CI: 0.118–0.879), while no significant differences were found with reference to the frequency of genotype TT. The frequency of genotype GG for rs893818 of *LOXL1* (*P* < 0.001, OR* *= 0.511, 95% CI: 0.358–0.729) and the frequency of genotype AA were both significantly higher in the XFS/XFG group compared to the control group (*P* < 0.001, OR* *= 0.095, 95% CI: 0.033–0.272). In addition, the frequency of genotype TT for rs41435250 of *LOXL1* was higher in XFS/XFG patients than in controls (*P*=0.003, OR* *= 3.902, 95% CI: 1.580–9.640), while no significant difference was found with reference to the frequency of genotype GG after adjusting for age and gender. All data are shown in Table 4.

Moreover, our results indicated that all MAFs were greater than 0.05, which further suggested that all SNPs were statistically significant (Table 5).

**Figure 1:**
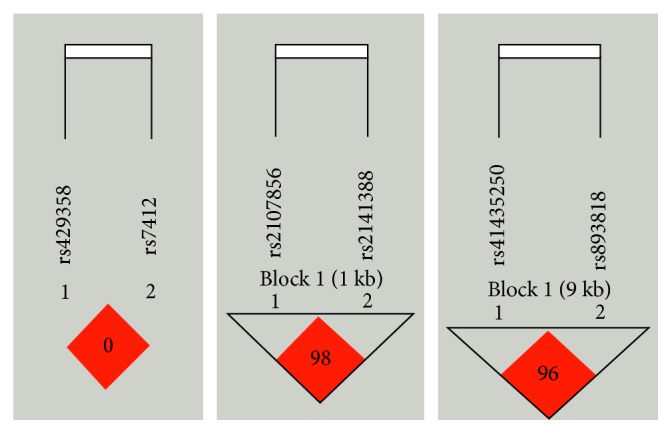
The genotyping graphs for LOXL1, APOE, and CNTNAP2.

After the study of alleles and genotypes, we screened out the LOXL1, APOE, and CNTNAP2 for the haplotype association analysis. The genotyping graphs for these SNPs are shown in Figure 1.

For the rs41435250 and rs893818 of *LOXL1*, three haplotypes were observed. As shown in Table 6, all haplotypes showed a significantly higher frequency in XFS/XFG patients than in controls: GA (*P* ≤ 0.001, OR* *= 0.417, 95% CI: 0.313–0.556), TG (*P* ≤ 0.001, OR* *= 1.772, 95% CI: 1.320–2.380), and GG (*P*=0.028, OR* *= 1.302, 95% CI: 1.030–1.648). Furthermore, after adjusting for age and gender, the similar data were obtained (Table 7): GA (*P* ≤ 0.001, OR = 0.400, 95% CI: 0.286–0.559), TG (*P*=0.001, OR = 1.769, 95% CI: 1.251–2.503), and GG (*P*=0.029, OR = 1.356, 95% CI: 1.032–1.782). We also observed three haplotypes for rs429358 and rs7412 of *APOE* and rs2107856 and rs2141388 of *CNTNAP2*; nevertheless, there was no connection between the case and control group.

## 4. Discussion

So far, numerous studies have focused on the polymorphisms of *LOXL1*. Our previous studies have shown that there were polymorphisms of *LOXL1* in different alleles and genotypes of different SNPs in XFS/XFG of different ethnic groups. In this study, we found two SNPs (rs41435250 and rs893818) of *LOXL1* that were polymorphic and associated with XFS/XFG. Meanwhile, we also examined other genes which were previously affirmed to have polymorphisms in XFS/XFG. We found that rs16958445 of *TBC1D21* and rs7137828 of *ATXN2* were significantly associated with XFS/XFG. Yet, three haplotypes for rs429358 and rs7412 of *APOE* and rs2107856 and rs2141388 of *CNTNAP2* had no connection between the case and control group.

As a result, *LOXL1* is still the susceptibility gene of XFS/XFG in Uygur populations. The rs1048661, rs3825942, rs2165241, rs4886467, rs4558370, rs4461027, rs4886761, rs16958477 [5, 6], and rs41435250 resulted to be risk factors, while rs893818 resulted to be a protective factor for XFS/XFG in the Uygur population.

Genes, such as *TBC1D21*, *ATXN2*, *APOE*, *CLU*, *AFAP1*, *TXNRD2*, *CACNA1A*, *ABCA1*, *GAS7*, and *CNTNAP2*, have been associated with glaucoma. In this study, we discovered that SNPs, rs16958445 of *TBC1D21* and the rs7137828 of *ATXN2*, had an important role in the pathogenesis of XFS/XFG in the Uygur population. Nonetheless, it is necessary to note that there may be other factors affecting the pathogenesis of XFS/G, which should be addressed by future studies.

In this research, we gathered a number of genes to study the polymorphisms of the special ethnic groups, thus providing valuable information and expanding the knowledge on the gene mechanism of XFS/XFG. Nonetheless, the current study has some limitations that should be pointed out. Although the patients were recruited from the three largest areas of Xinjiang, the sample representativeness may be somewhat inaccurate, which could be addressed by expanding the sample size and thus improving the accuracy. We found that multiple gene polymorphisms had an important role in the pathogenesis of the disorder in Uygur patients, but we cannot exclude the possibility that other additional genetic or environmental factors also participate in modifying the development of this disorder.

## Figures and Tables

**Table 1:** Baseline of the two groups.

	Case	Control	*t*	*P*
	*n*=216	*n*=297		
Age (years), mean ± SD	68.90 ± 8.47	62.46 ± 9.94	9.13	<0.001
Gender (M/F), *n* (%)	146 (67.59%)/70 (32.41%)	159 (53.54%)/138 (46.45%)	10.25	<0.001

M:male; F:female.

**Table 2:** Hardy–Weinberg equilibrium test of these SNPs.

GeneName	SNP	HWE_Case	HWE_Control	HWE
LOXL1	rs893818	0.255	0.310	0.324
LOXL1	rs41435250	0.497	1.000	0.505
TBC1D21	rs16958445	0.111	0.555	0.553
ATXN2	rs7137828	1.000	0.006	0.025
CNTNAP2	rs2107856	1.000	1.000	1.000
CNTNAP2	rs2141388	0.895	0.908	0.795
APOE	rs429358	0.484	0.755	0.253
APOE	rs7412	0.310	0.632	0.299
CLU	rs2279590	0.367	1.000	0.490
CACNA1A	rs4926244	0.074	0.616	0.099
ABCA1	rs2472493	0.895	0.646	0.862
GAS7	rs9897123	1.000	0.122	0.279
AFAP1	rs11732100	0.692	0.482	0.793
TXNRD2	rs35934224	0.005	1.000	0.101

Besides rs7137828 that deviated from the HWE in the control group and rs35934224 that deviated from the HWE in the case group, other SNPs were all in line with the HWE.

**Table 3:** Allele association analysis with these SNPs.

SNP	XFS/XFG	Control	*χ* ^2^	*P*	OR (95% CI)
TBC1D21					
rs16958445					
Allele					
G	432	583	3.025	0.082	1.369 (0.960–1.953)
A	70	69			

ATXN2					
rs7137828					
Allele					
T	429	531	3.272	0.070	0.7467 (0.544–1.025)
C	73	121			

APOE					
rs429358					
Allele					
T	452	587			
C	50	65	<0.001	0.996	0.999 (0.677–1.473)

rs7412					
Allele					
C	468	610			
T	34	42	0.051	0.822	1.055 (0.661–1.685)

CLU					
rs2279590					
Allele					
C	351	455			
T	151	197	0.002	0.961	0.994 (0.771–1.280)

AFAP1					
rs11732100					
Allele					
C	402	524			
T	100	128	0.015	0.903	1.018 (0.760–1.364)

TXNRD2					
rs35934224					
Allele					
C	446	574			
T	56	78	0.180	0.671	0.924 (0.642–1.331)

CACNA1A					
rs4926244					
Allele					
T	401	515			
C	101	137	0.138	0.710	0.947 (0.710–1.263)

ABCA1					
rs2472493					
Allele					
A	301	391			
G	201	261	<0.001	0.998	1.000 (0.789–1.269)

LOXL1					
rs41435250					
Allele					
G	379	552			
T	123	100	15.280	<0.001	1.791 (1.334–2.405)

rs893818					
Allele					
G	418	442			
A	84	210	35.780	<0.001	0.423 (0.318–0.563)

GAS7					
rs9897123					
Allele					
C	249	341			
T	253	311	0.827	0.363	1.114 (0.883–1.406)

CNTNAP2					
rs2107856					
Allele					
G	299	390			
T	203	262	0.008	0.930	1.011 (0.797–1.281)

rs2141388					
Allele					
C	301	391			
T	201	261	<0.001	0.998	1.000 (0.789–1.269)

G allele of rs41435250 of LOXL1 was the risk allele for the disorder. In contrast, G allele of rs893818 of LOXL1 was the protective allele for the disorder. Other alleles of SNPs showed no statistical significance.

**Table 4:** Genotype association analysis with these SNPs.

Gene/SNP	XFS/XFG	Control	p	OR (95% CI)	Adjusted-P	Adjusted-OR (95% CI)
TBC1D21						
rs16958445						
Genotype						
GG	189	259	0.532	1.138 (0.758–1.71)	0.114	1.465 (0.913–2.351)
GA	54	65	0.033	5.481 (1.151–26.110)	0.043	5.439 (1.053–28.090)
AA	8	2				

ATXN2						
rs7137828						
Genotype						
TT	183	224	0.705	0.929 (0.635–1.360)	0.955	1.013 (0.654–1.569)
TC	63	83	0.027	0.322 (0.118–0.879)	0.030	0.299 (0.100–0.891)
CC	5	19				

APOE						
rs429358						
Genotype						
TT	202	263	0.910	1.025 (0.673–1.560)	0.532	1.171 (0.714–1.920)
TC	48	61	0.727	0.651 (0.059–7.230)	0.773	0.697 (0.060–8.091)
CC	1	2				

rs7412						
Genotype						
CC	219	286	0.907	1.031 (0.619–1.717)	0.873	1.049 (0.585–1.882)
CT	30	38	0.790	1.306 (0.183–9.344)	0.887	1.172 (0.132–10.400)
TT	2	2				

CLU						
rs2279590						
Genotype						
CC	126	159	0.604	0.912 (0.644–1.292)	0.298	0.807 (0.538–1.209)
CT	99	137	0.760	1.094 (0.616–1.943)	0.171	1.586 (0.819–3.071)
TT	26	30				

AFAP1						
rs11732100						
Genotype						
CC	162	208	0.678	0.927 (0.649–1.324)	0.922	0.979 (0.650–1.477)
CT	78	108	0.442	1.412 (0.585–3.407)	0.576	0.711 (0.215–2.350)
TT	11	10				

TXNRD2						
rs35934224						
Genotype						
CC	203	252	0.118	0.709 (0.461–1.091)	0.175	0.710 (0.433–1.165)
CT	40	70	0.142	2.483 (0.737–8.362)	0.258	2.245 (0.553–9.114)
TT	8	4				

CACNA1A						
rs4926244						
Genotype						
TT	165	205	0.349	0.840 (0.584–1.209)	0.224	0.774 (0.512–1.170)
TC	71	105	0.684	1.165 (0.559–2.426)	0.826	0.901 (0.356–2.282)
CC	15	16				

ABCA1						
rs2472493						
Genotype						
AA	91	115	0.713	0.934 (0.650–1.343)	0.831	0.955 (0.626–1.457)
AG	119	161	0.888	1.036 (0.631–1.702)	0.906	0.966 (0.544–1.716)
GG	41	50				

LOXL1						
rs41435250						
Genotype						
GG	145	233	0.006	1.663 (1.158–2.388)	0.071	1.478 (0.966–2.259)
GT	89	86	0.003	3.902 (1.580–9.640)	0.003	5.276 (1.748–15.930)
TT	17	7				

rs893818						
Genotype						
GG	171	154	<0.001	0.511 (0.358–0.729)	<0.001	0.4449 (0.293–0.676)
GA	76	134	<0.001	0.095 (0.033–0.272)	<0.001	0.119 (0.039–0.356)
AA	4	38				

GAS7						
rs9897123						
Genotype						
CC	62	82	0.739	0.934 (0.625–1.396)	0.440	0.833 (0.523–1.325)
CT	125	177	0.335	1.263 (0.785–2.033)	0.808	0.935 (0.542–1.612)
TT	64	67				

CNTNAP2						
rs2107856						
Genotype						
GG	89	117	0.917	1.02 (0.709–1.467)	0.687	1.091 (0.715–1.665)
GT	121	156	0.947	1.017 (0.622–1.664)	0.537	1.194 (0.681–2.094)
TT	41	53				

rs2141388						
Genotype						
CC	91	118	0.981	0.996 (0.692–1.431)	0.772	1.064 (0.698–1.624)
CT	119	155	0.990	1.003 (0.614–1.639)	0.570	1.177 (0.672–2.060)
TT	41	53				

The genotypes AA for rs16958445 of TBC1D21 and GG/TT for rs41435250 of LOXL1 were risk genotypes for the disease. The genotypes CC for rs7137828 of ATXN2 and GG/AA for rs893818 of LOXL1 were protective genotypes for the disease.

**Table 5:** MAFs of these SNPs.

Gene	SNP	Ref allele	Alt allele	Case MAF	Control MAF	Total MAF
LOXL1	rs893818	G	A	0.167	0.322	0.255
LOXL1	rs41435250	G	T	0.245	0.153	0.193
TBC1D21	rs16958445	G	A	0.139	0.106	0.121
ATXN2	rs7137828	T	C	0.145	0.186	0.168
CNTNAP2	rs2107856	G	T	0.404	0.402	0.403
CNTNAP2	rs2141388	C	T	0.400	0.400	0.400
APOE	rs429358	T	C	0.100	0.100	0.100
APOE	rs7412	C	T	0.068	0.064	0.066
CLU	rs2279590	C	T	0.301	0.302	0.302
CACNA1A	rs4926244	T	C	0.201	0.210	0.206
ABCA1	rs2472493	A	G	0.400	0.400	0.400
GAS7	rs9897123	C	T	0.496	0.477	0.489
AFAP1	rs11732100	C	T	0.199	0.196	0.198
TXNRD2	rs35934224	C	T	0.112	0.120	0.116

All MAFs were greater than 0.05, which pointed out that all these SNPs were statistically significant.

**Table 6:** Haplotype association analysis between these SNPs.

Gene	Haplotype		Case (proportion)	Control (proportion)	*P* value	OR	95% CI
	rs41435250	rs893818					
LOXL1	G	A	83 (0.165)	210 (0.322)	≤0.001	0.417	0.313–0.556
	T	G	122 (0.243)	100 (0.153)	≤0.001	1.772	1.320–2.380
	G	G	296 (0.590)	342 (0.525)	0.028	1.302	1.030–1.648

	rs429358	rs7412					
APOE	T	C	418 (0.833)	545 (0.836)	0.884	0.977	0.715–1.336
	C	C	50 (0.100)	65 (0.100)	0.996	0.999	0.677–1.473
	C	T	34 (0.068)	42 (0.064)	0.822	1.055	0.661–1.685

	rs2107856	rs2141388					
CNTNAP2	T	T	201 (0.400)	261 (0.400)	0.998	1.000	0.789–1.269
	G	C	299 (0.596)	390 (0.598)	0.930	0.990	0.781–1.254

The haplotypes GG/TG/GA for the SNPs rs41435250 and rs893818 were significantly associated with XFS/XFG.

**Table 7:** Haplotype (adjusted) association analysis between these SNPs.

Gene	Haplotype		Case (proportion)	Control (proportion)	*P* value	OR	95% CI
	rs41435250	rs893818					
LOXL1	G	A	67 (0.155)	189 (0.319)	≤0.001	0.400	0.286–0.559
	T	G	101 (0.234)	89 (0.150)	0.001	1.769	1.251–2.503
	G	G	264 (0.611)	314 (0.530)	0.029	1.356	1.032–1.782

	rs429358	rs7412					
APOE	T	C	360 (0.833)	494 (0.834)	0.609	0.910	0.634–1.307
	C	C	42 (0.097)	57 (0.096)	0.643	1.113	0.707–1.753
	C	T	30 (0.069)	41 (0.069)	0.834	1.059	0.621–1.806

	rs2107856	rs2141388					
CNTNAP2	T	T	180 (0.417)	239 (0.404)	0.569	1.083	0.823–1.424
	G	C	250 (0.579)	352 (0.595)	0.523	0.915	0.696–1.202

The haplotypes GG/TG/GA for the SNPs rs41435250 and rs893818 were significantly associated with XFS/XFG.

## Data Availability

The data used to support the findings of this study are available from the corresponding author upon request.

## Abbreviations

XFS: Exfoliation syndrome
XFG: Exfoliation glaucoma
SNPs: Single-nucleotide polymorphisms
*LOXL1*: Lysyl oxidase-like 1 gene
APOE: Apolipoprotein E
CNTNAP2: Contactin-associated protein-like 2
TBC1D21: TBC1 domain family member 21
ATXN2: Autosomal-dominant ataxin 2
TXNRD2: Thioredoxin reductase 2
AFAP1: Actin filament-associated protein 1
ABCA1: ATP-binding cassette subfamily A member 1
GAS7: Growth arrest-specific 7
CACNA1A: Calcium voltage-gated channel subunit alpha1 A
CLU: Clusterin
DNA: Deoxyribonucleic acid
PCR: Photoconductive relay
HWE: Hardy–Weinberg equilibrium
OR: Odds ratios
CI: 95% confidence intervals.
